# Circular RNAs: characteristics, biogenesis, mechanisms and functions in liver cancer

**DOI:** 10.1186/s13045-021-01145-8

**Published:** 2021-08-30

**Authors:** Hao Shen, Boqiang Liu, Junjie Xu, Bin Zhang, Yifan Wang, Liang Shi, Xiujun Cai

**Affiliations:** 1 Zhejiang Provincial Key Laboratory of Laparoscopic Technology, Sir Run Run Shaw Hospital, Zhejiang University, Hangzhou, 310016 China; 2 Department of General Surgery, Sir Run Run Shaw Hospital, School of Medicine, Zhejiang University, Hangzhou, 310016 China; 3 Zhejiang Minimal Invasive Diagnosis and Treatment Technology Research Center of Severe Hepatobiliary Disease, Zhejiang University, Hangzhou, 310016 China; 4 Zhejiang Research and Development Engineering Laboratory of Minimally Invasive Technology and Equipment, Zhejiang University, Hangzhou, 310016 China; 5 Zhejiang University Cancer Center, Zhejiang University, Hangzhou, 310016 China

**Keywords:** CircRNAs, Characteristics, Biogenesis, Functions, Mechanisms, Liver cancer

## Abstract

**Background:**

Hepatocellular carcinoma (HCC) is one of the most common malignancies globally. Despite aggressive and multimodal treatment regimens, the overall survival of HCC patients remains poor.

**Main:**

Circular RNAs (circRNAs) are noncoding RNAs (ncRNAs) with covalently closed structures and tissue- or organ-specific expression patterns in eukaryotes. They are highly stable and have important biological functions, including acting as microRNA sponges, protein scaffolds, transcription regulators, translation templates and interacting with RNA-binding protein. Recent advances have indicated that circRNAs present abnormal expression in HCC tissues and that their dysregulation contributes to HCC initiation and progression. Furthermore, researchers have revealed that some circRNAs might serve as diagnostic biomarkers or drug targets in clinical settings. In this review, we systematically evaluate the characteristics, biogenesis, mechanisms and functions of circRNAs in HCC and further discuss the current shortcomings and potential directions of prospective studies on liver cancer-related circRNAs.

**Conclusion:**

CircRNAs are a novel class of ncRNAs that play a significant role in HCC initiation and progression, but their internal mechanisms and clinical applications need further investigation.

## Introduction

HCC was the sixth most common malignancy and the third leading cause of cancer-related death globally in 2020, accounts for 75–85% of primary liver cancer cases and has been ranked second in terms of mortality in men [1]. Chronic infection with hepatitis virus, alcohol abuse, metabolic syndrome related to diabetes and obesity are major risk factors for HCC [1]. However, despite the development of multimodal and advanced therapeutics, including surgical approaches and systemic drug treatment, the overall survival of HCC patients remains poor [2]. Researchers believe that this dilemma is caused by a lack of early diagnosis and the high HCC tumor heterogeneity [3]. Consequently, it is critical to deepen our understanding of HCC pathogenesis, tumor heterogeneity and mechanisms of resistance to systemic treatments and to further reveal potential biomarkers of HCC and identify novel therapeutic targets.

Emerging evidence indicates that noncoding RNAs (ncRNAs) are involved in many cellular biological and physiological processes and even pathological disease processes [4]. Among them, circRNAs are novel ncRNAs that are endogenous, abundant and stable in cells and have become a topic of intensive research in recent years [5]. Compared with canonical linear RNAs, this special type of ncRNA is generated from pre-mRNAs or other specific RNA molecules. [5]. CircRNAs were initially considered by-products or spliced intermediates of errant splicing; however, with the development of high-throughput sequencing techniques, many circRNAs have been found to be related to several diseases, including tumors [6–11]. Ongoing studies have revealed that circRNAs are able to regulate gene expression by modulating gene transcription and splicing, acting as miRNA sponges, interacting with proteins and translation of their own RNA sequence to produce polypeptides [5, 12–14]. Further research has shown that several specific circRNAs are dysregulated in HCC, and these functional molecules undoubtedly play a vital role in HCC progression and are regarded as potential diagnostic biomarkers or therapeutic targets [6, 15, 16]. However, the underlying mechanism of circRNAs in HCC initiation and development remains poorly understood. In this review, we summarize the characteristics, biogenesis, mechanisms and functions of circRNAs in HCC. Meanwhile, current shortcomings and possibilities for research in the field are broadly discussed.

## General features and formation of circRNAs

Various types of ncRNAs have been identified in the past two decades [4]. Among them, circRNA is a circular ncRNA that is mainly produced by special selective splicing and widely expressed in eukaryotic organisms [17], including *Drosophila*, mice and humans, and is also found in the hippocampus [5, 12]. Compared with linear RNAs, circRNAs are single-stranded, covalently closed circular transcripts without 5′ caps and 3′ tails [5, 12]. With the development of high-throughput sequencing technology and computational analysis, thousands of circRNAs have been discovered in organisms ranging from archaea to humans, and the abundance of some circRNAs is more than 10 times that of their corresponding linear RNAs [18].

Studies have revealed that circRNAs are mainly derived from pre-mRNAs and that the mechanism underlying circRNA formation differs from the standard splicing mechanism used to produce linear RNA [19]. CircRNAs are derived from a noncanonical form of alternative splicing called back-splicing [5, 12]. Despite the low efficiency of back-splicing compared to linear splicing, circRNAs present higher stability than their linear counterparts due to their covalently closed structure, which prevents exonuclease-mediated degradation [20].

**Fig. 1:**
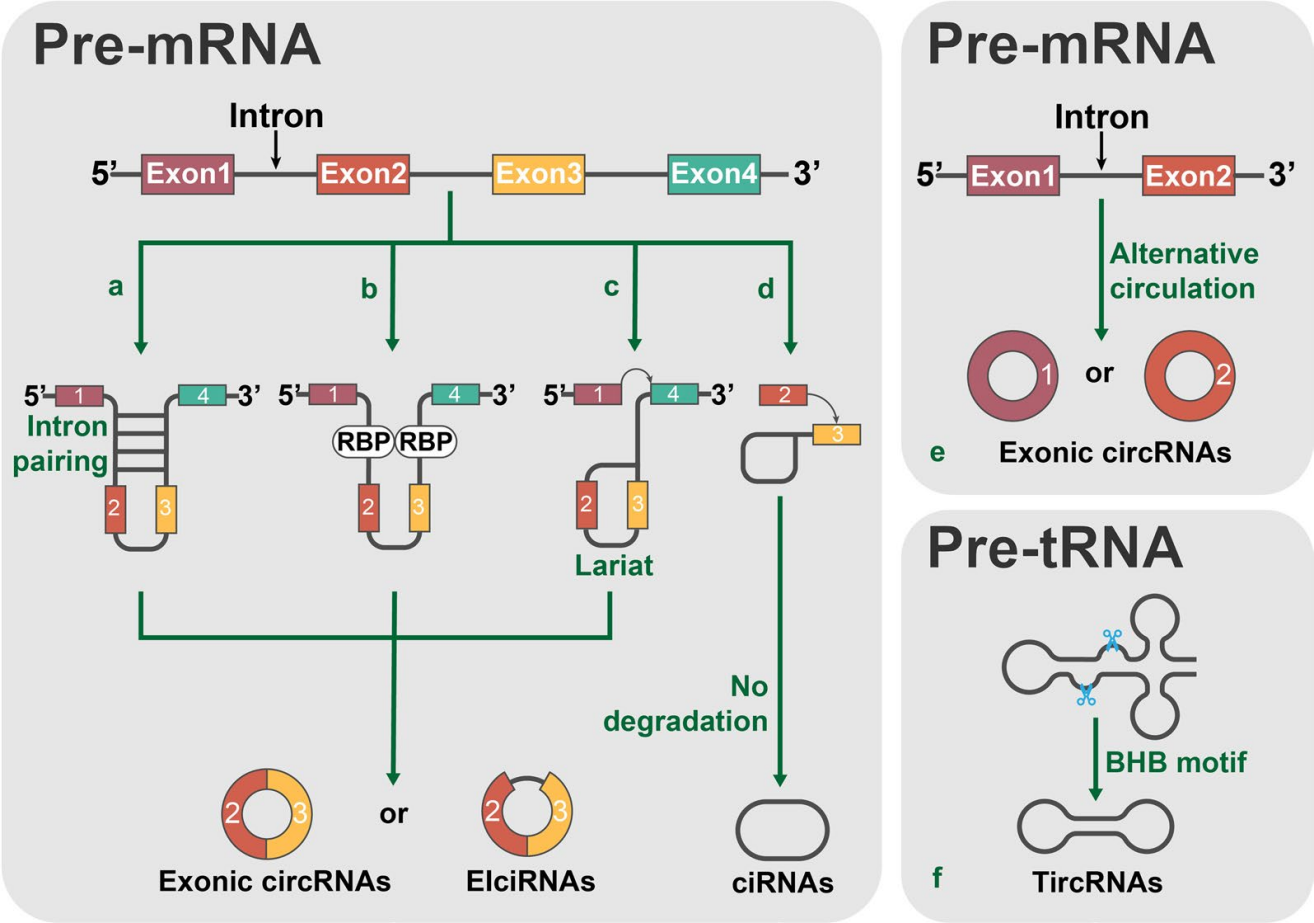
Biogenesis of circRNAs. Schematic showing the existing models of circRNA formation. **a** Intron pairing-driven circularization is accomplished by direct base pairing of the introns flanking complementary sequences or inverted repeats. **b** RBP-associated pairing-driven circularization is led by the close proximity of circRNA splice sites mediated by complementary base pairing of inverted repeats in the introns flanking the circRNA-forming exons. **c** Lariat-driven circularization facilitates the formation of an exon-containing lariat precursor starting from an exon-skipping event for efficient circle production. **d** CiRNA can be generated from intronic lariat precursors that escape the debranching step of canonical linear splicing. **e** Alternative back-splicing. **f** Formation of TricRNA. Through these different formation mechanisms, circRNAs can be divided into the following types: EcRNAs (exonic circRNAs), EIciRNAs (exon–intron circRNAs), ciRNAs (circular intronic RNAs) and TricRNAs (tRNA intronic circular RNAs)

The existing circRNA formation models mainly consist of the following: lariat-driven circularization, intron pairing-driven circularization, intronic circRNA (ciRNA) biogenesis, RBP-associated pairing-driven circularization and alternative back-splicing (Fig. 1) [5, 12, 15, 21–23]. Starting at an exon-skipping event, lariat-driven circularization helps to form an exon-containing lariat precursor for efficient circle production, while intron pairing-driven circularization is accomplished by direct base pairing of the introns flanking complementary sequences or inverted repeats [15, 24, 25]. Similarly, ciRNAs can be generated from intronic lariat precursors that escape the debranching step of canonical linear splicing [21]. RBP-associated pairing-driven circularization is led by the close proximity of circRNA splice sites mediated by complementary base pairing of inverted repeats in the introns flanking circRNA-forming exons [26]. This RBP-associated process requires the participation of RBPs, such as QKI, HNRNPL, FUS and MBL/MBNL1 [19, 26–28].

Interestingly, recent studies have provided evidence of a novel pattern: during pre-tRNA maturation, an intron-containing pre-tRNA can be cleaved by the tRNA splicing endonuclease (TSEN) complex at the bulge–helix–bulge (BHB) motif, and then, a single enzyme named RtcB ligase joins the exon halves and the intron ends to produce a mature tRNA and a circular intron RNA called tRNA intronic circular RNA (TricRNA) (Fig. 1) [16, 23].

Adenosine deaminase-acting on RNA (ADAR1) was found to be an RNA-editing enzyme and to play a suppressive role in circRNA formation. This function was correlated with its adenosine-to-inosine (A-to-I) editing process, which frequently occurs near the location of reverse complementary matches (RCMs), a conserved feature of circRNA biogenesis [29]. Interestingly, by utilizing a circRNA microarray survey, our group found that androgen receptor (AR) could diffusely suppress circRNA expression by upregulating ADAR1 p110 in HCC. This is currently a unique regulatory mechanism of circRNA biogenesis in HCC [30].

## Emerging technology in circRNA sequencing and detection

The emerging roles of circRNAs emphasize the importance of sequencing these circular transcripts. In the past decade, RNA sequencing (RNA-seq) technology has developed rapidly and has become an indispensable tool for analyzing the differential expression of genes and differential splicing events of mRNA at the transcriptome level [31].

Next-generation sequencing (also known as high-throughput sequencing) is currently the main way to sequence circRNAs. In next-generation sequencing, the coordination of gene cluster replication decreases with increasing read length, resulting in a decline in the quality of base sequencing. Therefore, next-generation sequencing is a high-throughput and short-read technique. Since most circRNAs are derived from exons, this alignment-based method is unable to distinguish circular reads from the overlapping regions of corresponding linear transcripts. The biggest drawback is that next-generation sequencing’s relatively short-read capacity considerably limits its detection ability in structural variant detection and genome assembly [32].

Emerging long-read sequencing technologies have become a powerful participant in genomics. Compared with short-read approaches, long-read technologies can generate continuous ultralong sequences directly from native DNA. However, oligo(dT) primers are not suitable to use for circRNA sequencing because of their lack of poly(A) tails, and as a result, long-read sequencing technology cannot be widely applied in circRNA studies [33].

Strikingly, a recent report presented a novel algorithm (CIRI-long) for detailed analysis of full-length circRNAs using nanopore sequencing technology. This technology utilized full-length circular reverse transcription (which was performed with random primers and SMARTer reverse transcriptase) to amplify circRNAs by producing long complementary DNA (cDNA) molecules containing multiple copies of full-length circRNA sequences. Then, a nanopore approach was used to directly sequence full-length circRNA sequences, and a specific algorithm was applied to quantify circRNA expression and recognize full-length mutant transcript sequences. The data showed that compared with Illumina RNA-seq (a platform for short-read, next-generation sequencing), nanopore sequencing can enhance the detection efficiency for circular reads (with a 20-fold increase) and provide a fivefold increase in identifying alternative circularization events, indicating its higher sensitivity for circular isoform identification. Furthermore, nanopore sequencing can recognize circRNAs at a relatively low abundance and capture nonclassical circRNAs more sensitively, such as a new type of intronic self-ligated circRNA [34].

Although many studies suggest that some circRNAs have the potential to be novel diagnostic or prognostic markers, the key problem is how to detect circRNAs in body fluids more efficiently. Very recently, our group reported a fully integrated electrochemical point-of-care testing (POCT) platform based on Au nanoflower (AuNF)/peptide nucleic acid (PNA)-modified carbon-fiber microelectrodes (CFMEs) [35]. The platform utilized PNA to recognize circRNA with high specificity and AuNFs to improve target-capturing efficiency at an ultralow level. Importantly, the analytical performance was verified in human serum samples, indicating the potential of this platform in clinical applications for HCC.

These new technologies provide more possibilities for the study of circRNAs. Undoubtedly, an increasing number of new technologies will emerge in the future, opening uncharted territory in circRNA research.

## CircRNAs profile in HCC

With the development of RNA sequencing technology, the whole genome transcriptional map of circRNAs in HCC has been reported in several studies [30, 36, 37].

To identify the circRNAs involved in HCC tumorigenesis, a recent study detected differential circRNA expression between HCC tissues and adjacent noncancerous liver (ANL) tissues. In this study, 13,686 distinct circRNAs were identified in total, excluding those with very low abundance, and the expression levels of the identified circRNAs were further analyzed. The results showed that 236 circRNAs were differentially expressed in HCC compared with matched ANL tissues, of which 108 were upregulated and 128 were downregulated [36].

Another study identified 220 circRNAs that were differentially expressed between patients who experienced postsurgical pulmonary metastasis and those who did not. Among the identified circRNAs, 144 were upregulated and 76 were downregulated [37].

HCC is characterized by a clear gender disparity, and AR is thought to be critical for this bias [38]. To verify the role of AR, our group used a circRNA microarray to analyze circRNA expression. The results identified a total of 508 dysregulated circRNAs in the AR-knockdown groups compared with the control groups. Among these circRNAs, 331 were upregulated, and 177 were downregulated [30]. These findings highlight the fact that circRNA profiles are widely dysregulated in HCC.

These sequencing and bioinformatics analysis statistics demonstrate the possible involvement of circRNAs in HCC tumorigenesis and development. Many studies have reported that circRNAs play an important role in HCC, although their specific functions and internal mechanisms are still under investigation [36, 37, 39–60] (Table 1).

**Table 1:** Overview of dysregulated circRNAs in hepatocellular carcinoma (HCC)

circRNAs	Dysregulation	Sponge targets	Downstream genes/proteins	Phenotype	References
circCDYL	Up	miR-892a miR-328-3p	HDGF/HIF1AN	Proliferation (+)	[39]
circSLC3A2	Up	miR-490-3p	PPM1F	Proliferation (+)	[40]
circFBLIM1	Up	miR-346	FBLIM1	Proliferation (+)	[41]
circHIPK3	Up	miR-124	AQP3	Proliferation (+)	[42]
circZNF566	Up	miR-4738-3p	TDO2	Proliferation (+)	[43]
circMAT2B	Up	miR-338-3p	PKM2	Proliferation (+); Metastasis (+)	[44]
circASAP1	Up	miR-326 miR-532-5p	MAPK1 CSF-1	Proliferation (+); Metastasis (+)	[37]
circDYNC1H1	Up	miR-140-5p	SULT2B1	Proliferation (+)	[45]
circARFGEF2	Up	miR-143-3p PCBP1	FOSL2 CD44v6	Proliferation (+); Metastasis (+)	[46]
circMAST1	Up	miR-1299	CTNND1	Proliferation (+)	[47]
circBACH1	Up	–	p27 HuR	Proliferation (+)	[48]
circRHOT1	Up	–	TIP60 NR2F6	Proliferation (+); Metastasis (+)	[49]
circβ-catenin	Up	–	β-catenin-370aa (circβ-catenin’s translation product)	Proliferation (+); Metastasis (+)	[50]
circUHRF1	Up	miR-449c-5p	TIM-3	Proliferation (+); Metastasis (+); Drug resistance (+); Immunity (−)	[51]
circSORE	Up	miR-103a-2-5p miR-660-3p	Wnt2b	Drug resistance (+)	[52]
circSORE	Up	–	YBX1	Drug resistance (+)	[53]
circMET	Up	miR-30-5p	CXCL10	Drug resistance (+); Immunity (−)	[54]
circUBAP2	Up	miR-194-3p	MMP9	Proliferation (+); Metastasis (+)	[59]
circPABPC1	Down	–	ITGB1	Proliferation (−); Metastasis (−)	[60]
circADD3	Down	–	EZH2 CDK1	Proliferation (−); Metastasis (−)	[55]
circMTO1	Down	miR-9	P21	Proliferation (−)	[56]
circSETD3	Down	miR-421	MAPK14	Proliferation (−)	[57]
circSMARCA5	Down	miR-17-3p miR-181b-5p	TIMP3	Proliferation (−)	[36]
circZKSCAN1	Down	–	FMRP CCAR1	Proliferation (−); Metastasis (−)	[58]

## Mechanisms of circRNAs in HCC

**Fig. 2:**
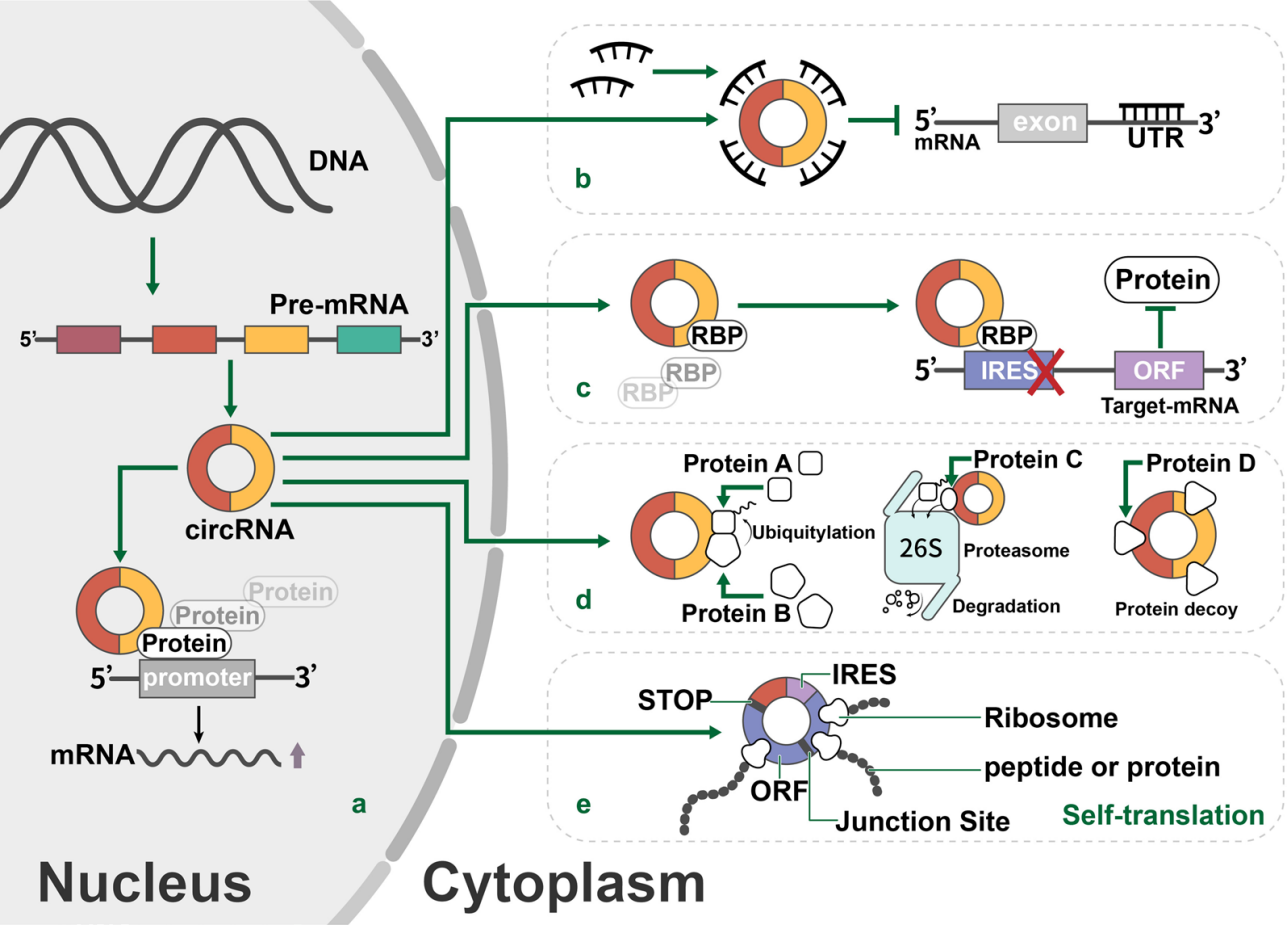
Mechanisms underlying circRNA function in HCC. This picture shows how circRNA performs its specific function in the nucleus and cytoplasm. **a** CircRNAs can regulate the transcription of their target genes. **b** CircRNAs can act as microRNA sponges to diminish microRNA function and protect target mRNAs from degradation. **c** CircRNAs can bind RNA-binding proteins (RBPs) to modulate the expression of related genes. **d** CircRNAs can interact with proteins to affect their structure and activity. **e** CircRNAs contain internal ribosome entry site (IRES) elements, and AUG sites may serve as templates to encode peptides or proteins

With further developments in the study of circRNAs, the mechanisms of circRNAs have been gradually revealed. Through pre-mRNA back-splicing, circRNAs gain high stability to exert their important regulatory functions. It is universally acknowledged that circRNAs play a vital role as competing endogenous RNAs (ceRNAs) to regulate downstream signaling pathways [61–63]. Meanwhile, numerous studies have verified that some circRNAs serve as scaffolds between different macromolecules to facilitate protein degradation, inhibit mRNA translation or initiate related gene transcription [48, 64, 65]. Similarly, circRNAs can also combine with RBPs to regulate gene expression [14]. Additionally, a specific circRNA named circβ-catenin, which is highly expressed in liver cancer tissues, can translate itself to promote liver cancer cell growth through a novel peptide; however, most circRNAs are regarded as ncRNAs (Fig. 2) [13, 50].

### MicroRNA sponging

The primary location of circRNAs is the cytoplasm, which is a precondition for them to exert posttranscriptional regulation functions [66]. Numerous studies have demonstrated that circRNAs can act as miRNA sponges to bind corresponding miRNAs and inhibit their function, thus regulating the translation or degradation of target mRNAs [63, 67]. CircRNAs possessing this property are defined as ceRNAs, which are found to contain competitive miRNA binding sites called miRNA response elements (MREs) [63]. The identification of ciRS-7 as a highly expressed circRNA in both human and mouse brains, with more than 70 conserved binding sites for miR-7, is noteworthy [62]. This famous circRNA behaves as a strong miR-7 sponge to decrease the level of miR-7 and indirectly activate the targets of miR-7 [62].

Ongoing investigations have reported that some circRNAs can function as miRNA sponges in HCC. CircSMARCA5 is a circRNA derived from exons 15 and 16 of the SMARCA5 gene and is downregulated in HCC [36]. In HCC cells, circSMARCA5 can serve as a sponge of miR-17-3p and miR-181b-5p to facilitate the expression of TIMP3, whereas downregulation of circSMARCA5 promotes HCC proliferation and metastasis [36]. Another study demonstrated that circASAP1 was overexpressed in high metastatic potential HCC and metastatic HCC and functioned as a ceRNA for miR-326 and miR-532-5p to regulate the expression of their direct targets MAPK1 and CSF-1, thereby promoting HCC cell proliferation and invasion and mediating tumor-associated macrophage infiltration [37]. Similarly, an oncogenic circRNA named circMAT2B was found to promote glycolysis and HCC malignancy by sponging miR-338-3p to activate the circMAT2B/miR-338-3p/PKM2 axis under hypoxia [44]. Recently, our laboratory reported that a sorafenib resistance-related circRNA named circSORE can sponge miR-103a-2-5p and miR-660-3p to competitively activate the Wnt/β-catenin pathway, thus inducing sorafenib resistance [52].

A large amount of evidence has confirmed the existence of the circRNA-miRNA pathway. However, it can be seen from the examples above that circRNAs usually sponge more than one miRNA to exert their function [36, 37, 52]. Therefore, for circRNAs with a single target miRNA or sponge site, their sponging function remains controversial. In addition, circRNAs are generally expressed at low levels and have relatively few binding sites for miRNAs in HCC, which may impair the efficiency of miRNA sponging [12]. We could also use the copy number ratio between circRNA and miRNA to evaluate the reliability of this specific function [16]. In summary, the idea of regulating the stability and quantity of miRNAs by circRNAs and achieving a measurable phenotype should be considered with caution.

### CircRNA–RBP interactions

RBP is a general term for a group of proteins possessing RNA recognition motifs that function by binding RNA to regulate its metabolic processes [68]. RBPs play an indispensable role in the maturation, transport, localization and translation of RNA [69]. RBP deficiency or dysfunction can induce various diseases. In recent years, a deeper understanding of the molecular mechanisms underlying RBP functions has promoted the development of new treatments for malignancies.

Several groups have reported that circRNAs may combine with RBPs to regulate gene expression [14, 19, 70, 71]. A recent investigation demonstrated that circBACH1 was significantly upregulated in HCC tissues and interacted with HuR (an extensively studied RBP that can inhibit p27 translation via an interferon-responsive sequence element in the p27 5′-untranslated region) to promote HuR translocation and facilitate its accumulation in the cytoplasm, thereby downregulating p27 expression [48, 72, 73]. Another notable circRNA called circZKSCAN1 was found to be closely related to malignancy and the overall survival rate of patients with HCC. Previous studies have shown that FMRP is highly expressed in HCC cells and acts as an RBP to modulate the translation of its target mRNAs [74]. Current studies have revealed that circZKSCAN1 plays a suppressive role by competitively binding FMRP and thus blocking the interaction between FMRP and CCAR1 mRNA, resulting in inhibition of the Wnt signaling pathway [58].

As mentioned above, circRNAs can physically combine with specific RBPs to facilitate or inhibit their function. Interestingly, one study revealed that a specific circRNA can alter the subcellular localization of its target RBP, providing a novel pattern for RBPs to perform specific functions under unusual cellular localization [48]. However, the interaction between circRNAs and RBPs in HCC remains unclear and requires further investigation. Considering this situation and according to our experience, we suggest utilizing RBP immunoprecipitation and circRNA sequencing to screen RBP-related circRNAs with potential regulatory functions in HCC.

### Protein scaffolding

Emerging evidence has demonstrated that some circRNAs may act as dynamic scaffolding molecules to regulate protein functions. Ongoing studies have shown that circRNAs can bind corresponding proteins in specific subcellular locations and facilitate the colocalization of relevant proteins, which may modulate protein–protein interactions [75].

An outstanding study revealed that circACC1 combines with the regulatory β and γ subunits of AMPK to form a ternary complex and thereby facilitates the stabilization and activity of the AMPK holoenzyme [76]. In HCC, a series of investigations found that circAMOTL1 can combine with c-myc, STAT3, PDK1 and AKT1 to promote their translocation to the nucleus and thereby modulate the expression of their target genes [77–79]. Meanwhile, a notable investigation reported that circRHOT1 could recruit TIP60 to the NR2F6 promoter and initiate NR2F6 transcription, resulting in HCC progression [49].

Recently, our laboratory demonstrated that circSORE could bind the master oncogenic protein YBX1 to prevent YBX1 nuclear interaction with the E3 ubiquitin ligase PRP19 and inhibit PRP19-mediated YBX1 degradation, thereby inducing sorafenib resistance [53]. In contrast, circADD3 was suggested to strengthen the interaction between CDK1 and EZH2 to facilitate EZH2 ubiquitination and subsequent degradation, indicating that circADD3 functions as a protein scaffold in CDK1-mediated EZH2 ubiquitination and ultimately restrains HCC metastasis [55]. Additionally, our group reported that circPABPC1 could exert a critical tumor-suppressive function by directly feeding ITGβ1, a classic membrane protein, to the proteasome for ubiquitin-independent degradation in HCC [60]. This unexpected finding should have a significant impact on our understanding of substrate recognition and protein degradation by the proteasome.

All these circRNAs serve as protein scaffolds. However, they exhibit different biological functions, suggesting that circRNAs can play different roles as protein scaffolds. Recent years have witnessed excellent progress in understanding of this novel mechanism in HCC, to which our group has made a contribution [60]. Given the high stability of circRNAs, we believe that circRNAs can act as protein scaffolds to exert their regulatory functions in a recycling manner, even if they are of low abundance.

### Translation into proteins

CircRNAs are regarded as ncRNAs due to the lack of essential components for cap-dependent translation, including the 5′ cap and the poly(A) tail. However, emerging evidence indicates that circRNAs may have the potential to encode proteins in a cap-independent manner [80]. circRNAs possessing internal ribosome entry sites (IRESs) in their sequence or N(6)-methyladenosine (m6A) in their 5' UTR may serve as translation templates to encode proteins [81, 82].

CircZNF609 is one of the few endogenous circRNAs that have been reported to act as translation templates. Structurally, circZNF609 contains a 753-nt open reading frame (ORF) spanning from the start codon of the host gene to a stop codon created 3 nt after the splice junction [83]. Mechanistically, the translation of circZNF609 occurs in a splicing-dependent and cap-independent manner, thus providing an example of circRNAs that have the potential to encode proteins. On the other hand, circZNF609 was reported to play a vital role in myogenesis and regulate myoblast proliferation [83]. However, the relationship between the circZNF609-induced phenotype and its protein-coding ability was not determined. Thus, whether this translation product has a functional role in biological processes is still unclear.

Recently, a novel circRNA, circβ-catenin, was identified as a protein-encoding circRNA whose translation can promote HCC cell growth through activation of the Wnt pathway [50]. The study showed that circβ-catenin was upregulated in liver cancer tissues and was able to encode a novel β-catenin isoform called β-catenin-370aa [50]. β-Catenin is well known as an oncogenic transcription factor that can activate the Wnt/β-catenin pathway to induce liver cancer progression [84]. Further studies revealed that β-catenin-370aa can act as a decoy for GSK3β to protect β-catenin from GSK3β-mediated degradation and thus indirectly promote liver cancer growth [50].

These results broaden our understanding of the human proteome. Currently, there is only one report on the translational function of circRNAs in HCC, indicating that the internal mechanisms of encoding circRNAs lack in-depth study. Our group suggests using a bioinformatics database to predict whether ORFs, IRESs or m6A modification sites exist in candidates. CircRNAs possessing these basic conditions may have the potential to encode proteins, which requires subsequent experimental analysis for further verification. The abundance and subcellular localization of circRNAs may lead to inferior translation efficiency compared with their cognate mRNAs. Hence, it remains controversial whether circRNA-derived proteins could achieve observable effects on the development of HCC [83].

## Role of circRNAs in HCC

**Fig. 3:**
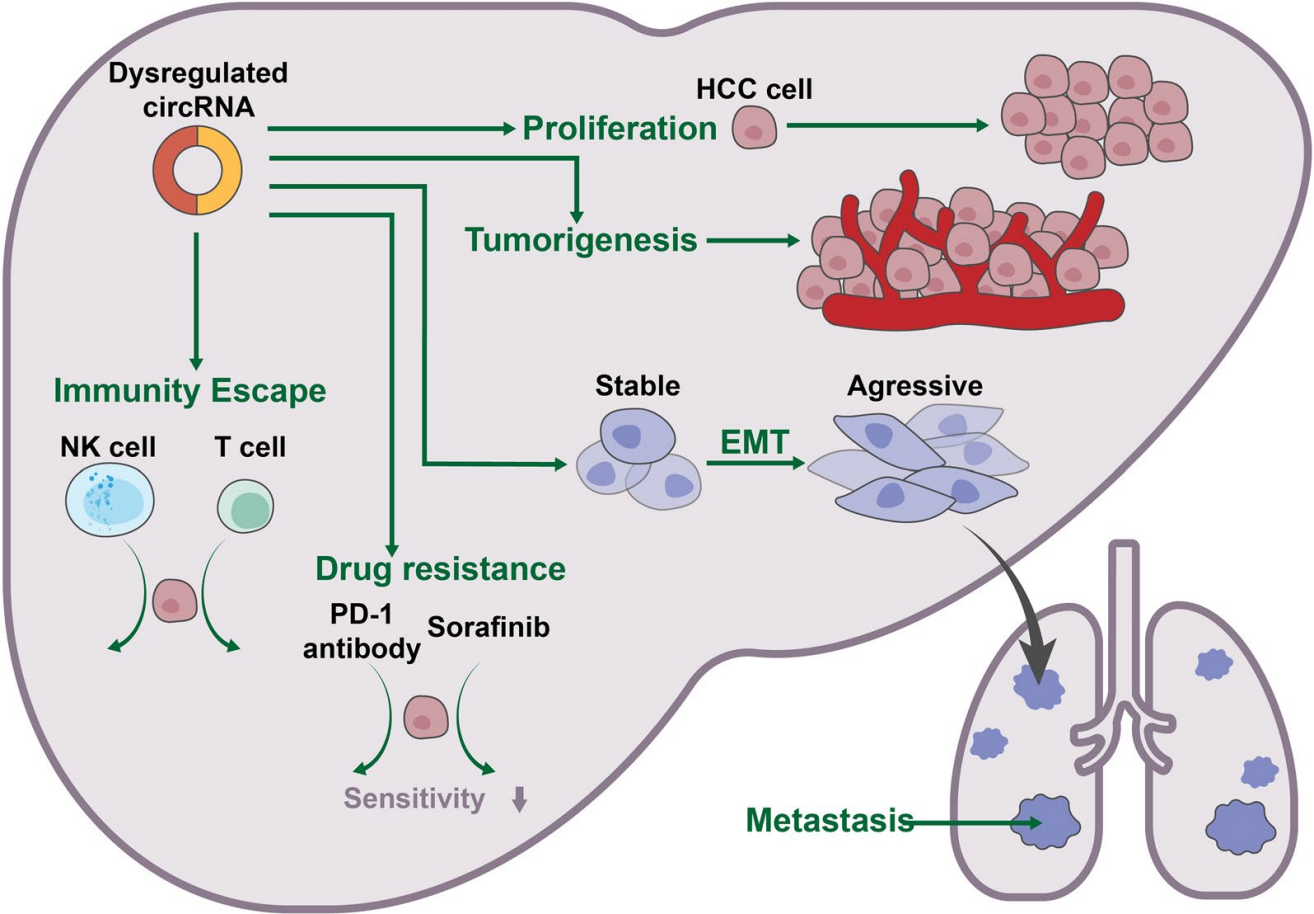
The many roles of circRNAs in HCC. CircRNAs can affect cell proliferation and tumorigenesis in HCC progression. CircRNAs are involved in the epithelial-to-mesenchymal transition (EMT) process, exerting important functions in HCC cell invasion and metastasis. CircRNAs can also affect HCC development and prognosis by regulating the immune system. Some circRNAs may even play a vital role in the induction and maintenance of drug resistance during HCC drug treatment

Emerging evidence has indicated that circRNAs play vital roles in HCC tumorigenesis and progression and are involved in cell proliferation, tumor metastasis, immune escape and drug resistance (Fig. 3) [36, 37, 44, 51–53]. In the following section, we will describe the roles of circRNAs in regulating these cellular processes.

### Cell proliferation and tumorigenesis

Investigations have reported that several circRNAs are expressed at an extremely high level in the early stages of HCC, suggesting that these specific circRNAs may promote tumorigenesis. A previous study demonstrated that circCDYL can regulate tumorigenesis by promoting the stem-like properties of HCC cells and showed that circCDYL-transduced HCC cells present a higher expression level of stem cell-associated genes with a markedly increased percentage of EpCAM + cells. Moreover, overexpression of circCDYL increased the expression of Ki-67 and alpha-fetoprotein (AFP), indicating that circCDYL promotes the malignant proliferation of HCC cells [39]. Another study unambiguously revealed that circCSNK1G1 could modulate the downstream genes TRAF6 and MAPK11, which are correlated with the MAPK signaling pathway and related to HCC tumorigenesis [85, 86]. CircSMARCA5, which has been strongly verified to be associated with HCC progression both in vitro and in vivo, simultaneously binds miR17-3p and miR-181b-5p to protect TIMP3 from downregulation, thus suppressing HCC proliferation [36].

On the other hand, emerging evidence indicates that circRNAs can regulate tumor cell apoptosis through specific mechanisms [87]. Apoptosis is highly restricted or inhibited in tumor cells through activation of antiapoptotic components, such as Bcl-2 or suppression of proapoptotic factors, including PUMA and BAX [65]. One study showed that circFoxo3 can bind p53 and MDM2 to repress p53 levels and increase Foxo3 levels, resulting in upregulation of PUMA and promotion of apoptosis [65, 88]. CircMBNL3, which is significantly downregulated in HCC tissues, acts as a ceRNA to inhibit proliferation and facilitate apoptosis in HCC by sponging miR-1307 [89].

Hence, the important role played by circRNAs in cell proliferation and tumorigenesis is becoming even clearer, providing insights for a deeper understanding of HCC pathogenesis.

### Cell migration and invasion

Epithelial-to-mesenchymal transition (EMT) refers to a biological process by which epithelial cells are transformed into cells with a mesenchymal phenotype, and EMT plays a vital role in the ability of malignant tumor cells derived from epithelial cells to obtain characteristics that enable invasion and metastasis [90]. Mechanistically, laminin-5 and TGF-β cooperatively induce EMT in HCC, and TGF-β has been identified as a crucial inducer of EMT and represents a vital EMT pathway leading to HCC progression [91]. Accumulating studies have revealed that EMT progression is associated with EMT transcription factors (e.g., Snail, Twist and ZEB1) and signaling pathways, including the TGF-β/Smad, Wnt/β-catenin and Hedgehog signaling pathways [90, 91].

Recent advances have shown that some EMT-related circRNAs can affect the EMT process in HCC. For instance, circARFGEF2 was found to be overexpressed in portal vein tumor thrombus (PVTT) and HCC tissues and to promote the EMT process via a miR-143-3p/FOSL2 axis and PCBP1/CD44v6 axis (FOSL2 is an EMT-related stimulator, and CD44v6 is an EMT-activating gene), leading to a remarkable acceleration in intrahepatic and pulmonary metastasis [46]. Moreover, circMTO1 is a remarkable tumor suppressor that represses HCC progression, and silencing of circMTO1 in HCC was found to downregulate the expression of p21, a target of oncogenic miR-9, thus promoting HCC cell invasion [56]. As previously mentioned, our group demonstrated that circPABPC1 can directly induce ubiquitin-independent proteasomal degradation of ITGβ1, inhibiting HCC tumor adhesion and metastasis [60].

Consequently, circRNAs are considered key factors with well-characterized functions in regulating the EMT process, cancer cell invasion and metastasis in HCC.

### Immunity escape

Immune dysfunction plays an important role in HCC development. Experimental evidence has shown that the circRNA expression profile changes during viral infection, which can regulate the function of the immune system [92]. Subsequent evidence indicated that circRNAs also have a regulatory effect on the immune system in HCC [51, 54].

For instance, a recent study found that HCC cells secrete circUHRF1 through exosomes and that circUHRF1 can inhibit NK cells from secreting IFN-γ and TNF-α by upregulating the expression of TIM-3 in NK cells. This study showed that the level of plasma exosomal circUHRF1 was negatively correlated with the infiltration level of NK cells in tumors [51]. Researchers have even proposed the hypothesis that circUHRF1 may promote the development of resistance to PD1 immunotherapy in HCC patients, although evidence is still insufficient. Another study reported that circMET is overexpressed in HCC cells and that a high level of circMET could facilitate CXCL10 degradation through the miR-30-5p/Snail/DPP4 axis, thereby enhancing immunosuppression and promoting HCC development [54].

These examples confirm that circRNAs can affect HCC development and prognosis by regulating the immune system of HCC patients, which prompted us to wonder whether circRNAs will become an ideal immunotherapy target in the future.

### Drug resistance

Statistics indicate that most HCC patients are diagnosed at an advanced stage. At present, the main drug treatment for unresectable HCC includes chemotherapy (e.g., cisplatin and 5-fluorouracil) and targeted therapy (e.g., sorafenib and monoclonal antibodies to either PD-1 or PD-L1), but conventional systemic chemotherapy lacks survival benefits.

Currently, multikinase inhibitors, monoclonal antibodies and immune checkpoint inhibitors are the main targeted molecular therapies approved for treatment of advanced-stage HCC; however, the effects of these treatments are not satisfactory [93, 94]. The main problem is that drug resistance is inevitable and emerges early in HCC. A previous study reported that sorafenib resistance was usually observed within 6 months of HCC treatment, and a similar situation was observed with anti-PD1 therapy [95, 96].

Increasing evidence has indicated that ncRNAs are critical for the development of sorafenib resistance in HCC [97, 98]. Our group revealed that miR-378a-3p can inhibit HCC sorafenib resistance by targeting IGF1R [99]. Another study demonstrated that the lncMALAT1/miR-140-5p/Aurora-A axis might regulate sorafenib resistance in HCC [100].

Meanwhile, ongoing studies have shown that circRNAs play a vital role in the development of drug resistance [7]. Our group has made continuous progress in this field and found that circSORE participates in the process of HCC sorafenib resistance [52, 53]. Additionally, circFN1 was reported to induce HCC sorafenib resistance by sponging miR-1205 and modulating E2F1 expression [101].

In summary, we must further clarify the mechanism underlying drug resistance and explore how circRNAs function in resistance to molecular targeted drugs. Identifying a specific circRNA that could be used as a new therapeutic target to avoid drug resistance would be an important step forward.

## Clinical application of circRNAs

### Diagnostic biomarkers

“Secondary prevention” (early detection, early diagnosis and early treatment) can improve the prognosis of HCC patients. The poor prognosis of most patients with HCC is due to the diagnosis of advanced HCC, and the reaction of advanced HCC to all treatment regimens is not satisfactory [102]. At present, the canonical biomarker used for early diagnosis of HCC is AFP; however, the sensitivity and specificity of this marker are not ideal [103]. Therefore, we urgently need to find new diagnostic biomarkers that can be used to detect and diagnose HCC at an earlier stage and interfere with HCC progression earlier to improve the prognosis of HCC patients. For instance, circCDYL has been shown to be highly and specifically expressed in early-stage HCC. Logistic regression and ROC curve analysis showed that assessment of circCDYL combined with assessment of HDGF and HIF1AN levels could distinguish early HCC from paracarcinoma tissues, which may provide a promising early diagnostic biomarker for HCC [39].

Emerging evidence has demonstrated that circRNAs hold great potential as a novel attractive class of diagnostic biomarkers for HCC due to their resistance to RNase R digestion and their stability during circulation. Recent studies have indicated that circRNAs can not only be detected in tumor tissues but also in exosomes, blood, saliva and urine [104–107]. The aberrant expression of circRNAs in body fluids from HCC patients makes them ideal noninvasive biopsy biomarker candidates.

### Prognostic biomarkers

CircRNAs have gradually been recognized as possible prognostic biomarkers. For instance, circSLC3A2, which is elevated in HCC tissues, plays an oncogenic role by sponging miR-490-3p to modulate PPM1F expression and could serve as a prognostic biomarker due to its positive correlation with poor survival in patients with HCC [40]. In contrast, another study found that the level of circNFATC3 is positively correlated with NFATC3 and that overexpression of NFATC3 predicts a better prognosis; thus, circNFATC3 might be a biomarker of good prognosis for HCC patients [108]. Additionally, our group found that circUBAP2 functions as a ceRNA for miR-194-3p to promote MM9-mediated oncogenic effects in HCC, indicating the great potential of circUBAP2 as a promising prognostic biomarker [59].

However, the clinical feasibility of using circRNAs still needs to be verified in a large cohort of HCC patient samples. Meanwhile, inventing efficient circRNA detection methods, such as the POCT platform, is also of great significance.

### Therapeutic targets

Recently, an increasing number of studies have focused on the significance of circRNAs in HCC and their correlation with HCC tumorigenesis and development, and some authors have proposed that circRNAs have the potential to serve as therapeutic targets in HCC (Table 2). For example, circMAT2B is involved in HCC glucose metabolism reprogramming and malignancy, and a previous study demonstrated that under hypoxic conditions, circMAT2B can induce HCC progression by enhancing glycolysis via activation of the circMAT2B/miR-338-3p/PKM2 axis, which may provide a therapeutic target for HCC [44].

**Table 2:** Potential application of circRNAs in HCC

circRNA	Stage	Functional mechanism	Potential application	References
cSMARCA5	Prediction	cSMARCA5 could sponge miR-17-3p and miR-181b-5p to upregulate TIMP3	1. A potential diagnostic biomarker in HCC (AUC values of 0.938 and 0.862) 2. A potential prognostic biomarker in HCC	[36]
circPABPC1	Pre-clinic	AR/ADAR1/CircPABPC1 axis	Potential therapeutic value for HCC through reactivation of this circular RNA	[30]
circPABPC1	Pre-clinic	circPABPC1 physically links ITGB1 to the 26S proteasome for ubiquitination-independent degradation	Potential therapeutic value for HCC treatment (technically possible with RNA-delivering methods)	[60]
circMAT2B	Pre-clinic	circMAT2B/miR-338-3p/PKM2 axis	1. A potential therapeutic target for HCC 2. A potential prognostic biomarker in HCC	[44]
circCDYL	Pre-clinic	circCDYL/miR-892a/HDGF circCDYL/miR-328-3p/HIF1AN	1. Promising biomarkers (combined with HDGF and HIF1AN) for surveillance in early stages of HCC (AUC values of 0.73, while the AUC of AFP was low at 0.59) 2. Potential therapeutic value in early treatment of HCC	[39]
circSETD3	Pre-clinic	circSETD3/miR-421/MAPK14 axis	1. A prognostic biomarker in patients with HCC who have received curative liver resection 2. A potential therapeutic target for HCC	[57]
circMTO1	Pre-clinic	circMTO1/miR-9/p21 axis	1. A prognostic biomarker for HCC patients 2. A potential target in HCC treatment	[56]
circFBXO11	Pre-clinic	circFBXO11/miR-605/FOXO3/ ABCB1 axis	1. A potential diagnostic biomarker in HCC 2. A potential therapeutic target for overcoming oxaliplatin resistance in HCC	[121]
circSORE	Pre-clinic	circSORE binds to YBX1 protein to prevent its PRP19-mediated degradation	1. A promising biomarker (exosome-carrying-circRNA-SORE) for early detection 2. Potential therapeutic value of circSORE siRNA for overcoming sorafenib resistance 3. Sorafenib resistance testing	[53]
circUHRF1	Pre-clinic	circUHRF1/miR-449c-5p/TIM-3 axis	1. Exosomal circUHRF1 provides a potential therapeutic value for overcoming anti-PD1 resistance in HCC 2. Anti-PD1 resistance testing	[51]
circFN1	Pre-clinic	circFN1/miR-1205/E2F1 axis	1. The circFN1/miR-1205/E2F1 signaling pathway may provide novel strategies to overcome sorafenib resistance in HCC 2. Sorafenib resistance testing	[101]

Currently, the main treatment for high-grade (middle and advanced) HCC is chemotherapy and molecular targeted therapy; however, the development of resistance to HCC treatments is an unavoidable problem, including resistance to traditional chemotherapy and even first-line molecular targeted therapies, such as sorafenib [97]. As previously mentioned, our group showed that circSORE can induce sorafenib resistance by functioning as a ceRNA and a protein scaffold, indicating that circSORE might be a new therapeutic target to reduce sorafenib resistance [53]. In view of this, circRNAs have potential for use as new therapeutic targets to avoid drug resistance.

### Drug development

Recently, circRNA-based therapy has attracted increasing attention. Compared with other drugs, such as monoclonal antibodies or small molecule inhibitors, circRNA-based therapy has several advantages. First, circRNA drugs have a long half-life (usually weeks to months), while traditional therapeutic drugs are unstable once they enter the circulatory system; therefore, with circRNA drugs, the patient dosing frequency can be decreased compared with that for antibodies or small molecules [109]. Second, drug resistance induced by ABC transporters or epigenetic modifications is an inevitable dilemma in cancer treatment, but such issues have not been reported for circRNA-based therapy [110, 111]. Additionally, a previous study reported that multiple cell types in the liver generate different types of vesicles, such as exosomes and microvesicles, which can transport circRNAs to other targeted cells or organs [112]. This paracrine effect provides circRNA-based drugs with broader targets along the whole signaling pathway compared with traditional drugs. Meanwhile, recent advances in RNA delivery methods have made it possible to deliver therapeutic circRNAs to specific lesions [113, 114].

On the other hand, proteolysis-targeting chimeras (PROTACs) are a burgeoning and promising field and can modulate protein concentrations at a posttranslational level by coopting the ubiquitin–proteasome system [115]. As previously mentioned, circPABPC1 has a tumor-suppressive function and potential therapeutic value for HCC treatment. The properties of circPABPC1 in ITGβ1 regulation bear considerable similarity with those of PROTACs, although circPABPC1 can mediate ubiquitin-independent degradation [60]. In subsequent research, we made specific modifications to circPABPC1, which indicates the possibility of inducing specific degradation of other oncoproteins. Coincidentally, an American start-up company named Orna Therapeutics (ORNA) went public successfully and recently announced the start of a “circRNA therapy” project. As the first company to utilize circRNA technology in the development of new therapies, ORNA's circRNA therapy project is mainly dedicated to designing and delivering engineered circRNAs with therapeutic effects, indicating the promising potential of circRNA-based therapy.

Generally, whether circRNAs have clinical value merits further study and discussion. Although circRNAs have the potential to serve as diagnostic markers, prognostic markers, therapeutic targets or novel drugs, these roles are based only on theoretical assumptions and predictions; thus, more substantive studies are needed to verify these conjectures (Table 2).

## Perspectives

### Insights and limitations of current research

Substantial progress has been achieved in specific circRNAs. These findings not only reveal a previously unexpected complexity of cellular regulatory mechanisms but have also identified many circRNAs with important physiological and clinical significance. Although the progress is exciting, it has led to many more questions than answers, which require future investigation.

First, most studies have focused on a specific circRNA and its downstream mechanisms, while the upstream mechanisms remain largely unknown. For example, how exactly are circRNAs formed? Under what circumstances do pre-mRNAs form circRNAs rather than mRNAs? Are there any factors that are specifically required for back-splicing but not for splicing? How are circRNAs exported from the nucleus to the cytoplasm? These issues are rarely mentioned in most current studies. However, research groups have tried to answer these questions, and m^6^A modification of circRNAs has been proposed to facilitate the cytoplasmic export of circRNAs [116]. However, in general, these fields remain poorly understood.

Second, current biological tools and methods applied in circRNA-related studies still have some obvious shortcomings. Due to the high degree of sequence similarity between circRNAs and their cognate mRNAs, off-target effects of circRNA knockdown are difficult to avoid or completely eliminate. Similarly, the same dilemma has been observed in circRNA FISH and circRNA pull-down experiments. To successfully overexpress circRNAs, special sequence elements that promote circularization are usually inserted at both ends of the linear circRNA sequence. Inevitably, a large number of “linear circRNAs” will be produced due to the low efficiency of circularization. Whether this by-product will affect experiments remains to be further studied. Furthermore, the CRISPR-Cas9 system may be restricted in knockout studies of circRNAs because deletion of the whole genome sequence could disturb the expression of cognate mRNAs and impact specific biological functions. These challenges highlight the need for more advanced technologies for circRNA interference, detection and other functional studies. Very recently, Chen and colleagues reported that the CRISPR-Cas13 technique combined with guide RNA targeting sequences spanning junction sites featured in circRNAs can be applied to knock down circRNAs. Furthermore, compared with shRNA-mediated knockdown, RfxCas13d–BSJ-gRNA-mediated circRNA knockdown showed higher efficiency and specificity and lower rates of off-target effects on cognate mRNAs [117].

Finally, the number of circRNAs that truly have cellular and physiological functions remains to be explored. In particular, our research has shown that the expression of circRNAs in HCC is widely reduced, suggesting that low expression levels will lead to nonfunctionalization [30]. Given the low abundance and relatively few miRNA binding sites of most circRNAs, how do they effectively sponge miRNAs [118]? The most striking example in this category is CDR1as, which contains more than 70 conserved binding sites for miR-7 and is abundantly expressed in mammalian brains [62, 119]. If the two conditions mentioned above are not met, the idea of sponging miRNAs by circRNAs and achieving measurable effects should be considered with caution. Hence, we should consider the characteristics of circRNAs when investigating their downstream mechanism. For instance, circRNAs with high abundance, various sponge sites and multiple target miRNAs may function as miRNA sponges; circRNAs with low abundance, a small size and high stability may serve as protein scaffolds; RBP immunoprecipitation and circRNA sequencing can be used to detect RBP-related circRNAs; and the existence of ORFs, IRESs or m6A modification sites on circRNAs suggests translational potential.

### Innovative suggestions for future research

In recent years, an increasing number of studies have begun to focus on circRNAs; thus, circRNA-related research is progressing at a steady and fast pace. In the following section, we propose several challenging and innovative future research directions in the field of circRNAs.

First, the current research remains at the cellular and animal levels, and how to facilitate translation toward clinical application will become a hot topic in the future. Because circRNAs are stable and have been detected in many types of body fluids, further studies are warranted to confirm their potential as biomarkers, therapeutic targets or novel drugs.

Second, whether a circRNA with relatively low abundance can achieve measurable effects remains controversial. Future studies should focus on certain types of circRNAs with the same properties instead of a specific circRNA. For example, a striking study reported that endogenous circRNAs with 16–26 bp that form imperfect RNA duplexes can act as inhibitors of double-stranded RNA (dsRNA)-activated protein kinase (PKR) related to innate immunity [120].

## Conclusions

Generally, HCC is a multistep, multistage and multifactor comprehensive hereditary malignancy, and the specific pathogenic mechanisms remain unclear. Despite the availability of a comprehensive treatment regimen, including surgery, chemotherapy, targeted therapy and immune therapy, the overall survival of patients with advanced HCC is still unsatisfactory. Notably, circRNAs are a novel class of ncRNAs that play a significant role in HCC initiation and progression. As described in this review, circRNAs are aberrantly expressed in HCC and associated with the clinicopathological features and prognosis of HCC patients. Unexpectedly, there is a high degree of heterogeneity among HCC-related circRNAs, and individual circRNAs may have prooncogenic or antioncogenic roles and function through various pathophysiological mechanisms. Given the stability and polyfunctionality of circRNAs, circRNAs might serve as diagnostic indicators, prognosis predictors, therapeutic targets or novel nucleic acid drugs for precise treatment of HCC, thus improving the quality of life and extending the survival of HCC patients.

## Data Availability

Not applicable.

## Abbreviations

HCC: Hepatocellular carcinoma
RBP: RNA-binding protein
HBV: Hepatitis B virus
HCV: Hepatitis C virus
ncRNA: Noncoding RNA
circRNA: Circular RNA
miRNA: MicroRNA
lncRNA: Long noncoding RNA
ciRNA: Intronic circRNA
TSEN: TRNA splicing endonuclease
BHB motif: Bulge–helix–bulge motif
TricRNA: TRNA intronic circular RNA
ANL: Adjacent noncancerous liver
AR: Androgen receptor
RNA-seq: RNA sequencing
cDNA: Complementary DNA
POCT: Point-of-care testing
AuNFs: Au nanoflowers
PNA: Peptide nucleic acid
CFME: Carbon-fiber microelectrode
ceRNA: Competing endogenous RNA
MREs: MiRNA response elements
IRES: Internal ribosome entry site
m6A: N(6)-methyladenosine
ORF: Open reading frame
AFP: Alpha-fetoprotein
EMT: Epithelial-to-mesenchymal transition
PVTT: Portal vein tumor thrombus
PROTAC: Proteolysis-targeting chimera
dsRNA: Double-stranded RNA

## References

[1] Sung H, Ferlay J, Siegel RL, Laversanne M, Soerjomataram I, Jemal A, Bray F (2021). Global cancer statistics 2020: GLOBOCAN estimates of incidence and mortality worldwide for 36 cancers in 185 countries. CA Cancer J Clin.

[2] Llovet JM, Kelley RK, Villanueva A, Singal AG, Pikarsky E, Roayaie S, Lencioni R, Koike K, Zucman-Rossi J, Finn RS (2021). Hepatocellular carcinoma. Nat Rev Dis Primers.

[3] Yang JD, Hainaut P, Gores GJ, Amadou A, Plymoth A, Roberts LR (2019). A global view of hepatocellular carcinoma: trends, risk, prevention and management. Nat Rev Gastroenterol Hepatol.

[4] Goodall GJ, Wickramasinghe VO (2021). RNA in cancer. Nat Rev Cancer.

[5] Chen LL (2020). The expanding regulatory mechanisms and cellular functions of circular RNAs. Nat Rev Mol Cell Biol.

[6] Wang M, Yu F, Li P (2018). Circular RNAs: characteristics, function and clinical significance in hepatocellular carcinoma. Cancers (Basel).

[7] Ma S, Kong S, Wang F, Ju S (2020). CircRNAs: biogenesis, functions, and role in drug-resistant Tumours. Mol Cancer.

[8] Tan S, Gou Q, Pu W, Guo C, Yang Y, Wu K, Liu Y, Liu L, Wei YQ, Peng Y (2018). Circular RNA F-circEA produced from EML4-ALK fusion gene as a novel liquid biopsy biomarker for non-small cell lung cancer. Cell Res.

[9] Jeck WR, Sharpless NE (2014). Detecting and characterizing circular RNAs. Nat Biotechnol.

[10] Guarnerio J, Bezzi M, Jeong JC, Paffenholz SV, Berry K, Naldini MM, Lo-Coco F, Tay Y, Beck AH, Pandolfi PP (2016). Oncogenic role of fusion-circRNAs derived from cancer-associated chromosomal translocations. Cell.

[11] Vo JN, Cieslik M, Zhang Y, Shukla S, Xiao L, Zhang Y, Wu YM, Dhanasekaran SM, Engelke CG, Cao X (2019). The landscape of circular RNA in cancer. Cell.

[12] Kristensen LS, Andersen MS, Stagsted LVW, Ebbesen KK, Hansen TB, Kjems J (2019). The biogenesis, biology and characterization of circular RNAs. Nat Rev Genet.

[13] Lei M, Zheng G, Ning Q, Zheng J, Dong D (2020). Translation and functional roles of circular RNAs in human cancer. Mol Cancer.

[14] Okholm TLH, Sathe S, Park SS, Kamstrup AB, Rasmussen AM, Shankar A, Chua ZM, Fristrup N, Nielsen MM, Vang S (2020). Transcriptome-wide profiles of circular RNA and RNA-binding protein interactions reveal effects on circular RNA biogenesis and cancer pathway expression. Genome Med.

[15] Yao R, Zou H, Liao W (2018). Prospect of circular RNA in hepatocellular carcinoma: a novel potential biomarker and therapeutic target. Front Oncol.

[16] Li J, Sun D, Pu W, Wang J, Peng Y (2020). Circular RNAs in cancer: biogenesis, function, and clinical significance. Trends Cancer.

[17] Zhang XO, Dong R, Zhang Y, Zhang JL, Luo Z, Zhang J, Chen LL, Yang L (2016). Diverse alternative back-splicing and alternative splicing landscape of circular RNAs. Genome Res.

[18] Gao Y, Shang S, Guo S, Li X, Zhou H, Liu H, Sun Y, Wang J, Wang P, Zhi H (2020). Lnc2Cancer 3.0: an updated resource for experimentally supported lncRNA/circRNA cancer associations and web tools based on RNA-seq and scRNA-seq data. Nucleic Acids Res.

[19] Ashwal-Fluss R, Meyer M, Pamudurti NR, Ivanov A, Bartok O, Hanan M, Evantal N, Memczak S, Rajewsky N, Kadener S (2014). circRNA biogenesis competes with pre-mRNA splicing. Mol Cell.

[20] Jeck WR, Sorrentino JA, Wang K, Slevin MK, Burd CE, Liu J, Marzluff WF, Sharpless NE (2013). Circular RNAs are abundant, conserved, and associated with ALU repeats. RNA.

[21] Zhang Y, Zhang XO, Chen T, Xiang JF, Yin QF, Xing YH, Zhu S, Yang L, Chen LL (2013). Circular intronic long noncoding RNAs. Mol Cell.

[22] Li Z, Huang C, Bao C, Chen L, Lin M, Wang X, Zhong G, Yu B, Hu W, Dai L (2015). Exon-intron circular RNAs regulate transcription in the nucleus. Nat Struct Mol Biol.

[23] Schmidt CA, Giusto JD, Bao A, Hopper AK, Matera AG (2019). Molecular determinants of metazoan tricRNA biogenesis. Nucleic Acids Res.

[24] Barrett SP, Wang PL, Salzman J (2015). Circular RNA biogenesis can proceed through an exon-containing lariat precursor. Elife.

[25] Lee Y, Rio DC (2015). Mechanisms and regulation of alternative pre-mRNA splicing. Annu Rev Biochem.

[26] Conn SJ, Pillman KA, Toubia J, Conn VM, Salmanidis M, Phillips CA, Roslan S, Schreiber AW, Gregory PA, Goodall GJ (2015). The RNA binding protein quaking regulates formation of circRNAs. Cell.

[27] Errichelli L, Dini Modigliani S, Laneve P, Colantoni A, Legnini I, Capauto D, Rosa A, De Santis R, Scarfo R, Peruzzi G (2017). FUS affects circular RNA expression in murine embryonic stem cell-derived motor neurons. Nat Commun.

[28] Fei T, Chen Y, Xiao T, Li W, Cato L, Zhang P, Cotter MB, Bowden M, Lis RT, Zhao SG (2017). Genome-wide CRISPR screen identifies HNRNPL as a prostate cancer dependency regulating RNA splicing. Proc Natl Acad Sci U S A.

[29] Ivanov A, Memczak S, Wyler E, Torti F, Porath HT, Orejuela MR, Piechotta M, Levanon EY, Landthaler M, Dieterich C, Rajewsky N (2015). Analysis of intron sequences reveals hallmarks of circular RNA biogenesis in animals. Cell Rep.

[30] Shi L, Yan P, Liang Y, Sun Y, Shen J, Zhou S, Lin H, Liang X, Cai X (2017). Circular RNA expression is suppressed by androgen receptor (AR)-regulated adenosine deaminase that acts on RNA (ADAR1) in human hepatocellular carcinoma. Cell Death Dis.

[31] Stark R, Grzelak M, Hadfield J (2019). RNA sequencing: the teenage years. Nat Rev Genet.

[32] Mardis ER (2008). Next-generation DNA sequencing methods. Annu Rev Genomics Hum Genet.

[33] Logsdon GA, Vollger MR, Eichler EE (2020). Long-read human genome sequencing and its applications. Nat Rev Genet.

[34] Zhang J, Hou L, Zuo Z, Ji P, Zhang X, Xue Y, Zhao F (2021). Comprehensive profiling of circular RNAs with nanopore sequencing and CIRI-long. Nat Biotechnol.

[35] Zhang B, Chen M, Cao J, Liang Y, Tu T, Hu J, Li T, Cai Y, Li S, Liu B (2021). An integrated electrochemical POCT platform for ultrasensitive circRNA detection towards hepatocellular carcinoma diagnosis. Biosens Bioelectron.

[36] Yu J, Xu QG, Wang ZG, Yang Y, Zhang L, Ma JZ, Sun SH, Yang F, Zhou WP (2018). Circular RNA cSMARCA5 inhibits growth and metastasis in hepatocellular carcinoma. J Hepatol.

[37] Hu ZQ, Zhou SL, Li J, Zhou ZJ, Wang PC, Xin HY, Mao L, Luo CB, Yu SY, Huang XW (2020). Circular RNA sequencing identifies CircASAP1 as a key regulator in hepatocellular carcinoma metastasis. Hepatology.

[38] Zhang H, Li XX, Yang Y, Zhang Y, Wang HY, Zheng XFS (2018). Significance and mechanism of androgen receptor overexpression and androgen receptor/mechanistic target of rapamycin cross-talk in hepatocellular carcinoma. Hepatology.

[39] Wei Y, Chen X, Liang C, Ling Y, Yang X, Ye X, Zhang H, Yang P, Cui X, Ren Y (2020). A noncoding regulatory RNAs network driven by circ-CDYL acts specifically in the early stages hepatocellular carcinoma. Hepatology.

[40] Wang H, Chen W, Jin M, Hou L, Chen X, Zhang R, Zhang J, Zhu J (2018). CircSLC3A2 functions as an oncogenic factor in hepatocellular carcinoma by sponging miR-490-3p and regulating PPM1F expression. Mol Cancer.

[41] Bai N, Peng E, Qiu X, Lyu N, Zhang Z, Tao Y, Li X, Wang Z (2018). circFBLIM1 act as a ceRNA to promote hepatocellular cancer progression by sponging miR-346. J Exp Clin Cancer Res.

[42] Chen G, Shi Y, Liu M, Sun J (2018). circHIPK3 regulates cell proliferation and migration by sponging miR-124 and regulating AQP3 expression in hepatocellular carcinoma. Cell Death Dis.

[43] Li S, Weng J, Song F, Li L, Xiao C, Yang W, Xu J (2020). Circular RNA circZNF566 promotes hepatocellular carcinoma progression by sponging miR-4738-3p and regulating TDO2 expression. Cell Death Dis.

[44] Li Q, Pan X, Zhu D, Deng Z, Jiang R, Wang X (2019). Circular RNA MAT2B promotes glycolysis and malignancy of hepatocellular carcinoma through the miR-338-3p/PKM2 axis under hypoxic stress. Hepatology.

[45] Wang ZY, Zhu Z, Wang HF, Qin B, Liu J, Yao XH, Li WC, Chen KS (2019). Downregulation of circDYNC1H1 exhibits inhibitor effect on cell proliferation and migration in hepatocellular carcinoma through miR-140-5p. Cell Physiol.

[46] Song LN, Qiao GL, Yu J, Yang CM, Chen Y, Deng ZF, Song LH, Ma LJ, Yan HL (2020). Hsa_circ_0003998 promotes epithelial to mesenchymal transition of hepatocellular carcinoma by sponging miR-143-3p and PCBP1. J Exp Clin Cancer Res.

[47] Yu X, Sheng P, Sun J (2020). The circular RNA circMAST1 promotes hepatocellular carcinoma cell proliferation and migration by sponging miR-1299 and regulating CTNND1 expression. Cell Death Dis.

[48] Liu B, Yang G, Wang X, Liu J, Lu Z, Wang Q, Xu B, Liu Z, Li J (2020). CircBACH1 (hsa_circ_0061395) promotes hepatocellular carcinoma growth by regulating p27 repression via HuR. J Cell Physiol.

[49] Wang L, Long H, Zheng Q, Bo X, Xiao X, Li B (2019). Circular RNA circRHOT1 promotes hepatocellular carcinoma progression by initiation of NR2F6 expression. Mol Cancer.

[50] Liang WC, Wong CW, Liang PP, Shi M, Cao Y, Rao ST, Tsui SK, Waye MM, Zhang Q, Fu WM, Zhang JF (2019). Translation of the circular RNA circbeta-catenin promotes liver cancer cell growth through activation of the Wnt pathway. Genome Biol.

[51] Zhang PF, Gao C, Huang XY, Lu JC, Guo XJ, Shi GM, Cai JB, Ke AW (2020). Cancer cell-derived exosomal circUHRF1 induces natural killer cell exhaustion and may cause resistance to anti-PD1 therapy in hepatocellular carcinoma. Mol Cancer.

[52] Xu J, Wan Z, Tang M, Lin Z, Jiang S, Ji L, Gorshkov K, Mao Q, Xia S, Cen D (2020). N(6)-methyladenosine-modified CircRNA-SORE sustains sorafenib resistance in hepatocellular carcinoma by regulating beta-catenin signaling. Mol Cancer.

[53] Xu J, Ji L, Liang Y, Wan Z, Zheng W, Song X, Gorshkov K, Sun Q, Lin H, Zheng X (2020). CircRNA-SORE mediates sorafenib resistance in hepatocellular carcinoma by stabilizing YBX1. Signal Transduct Target Ther.

[54] Huang XY, Zhang PF, Wei CY, Peng R, Lu JC, Gao C, Cai JB, Yang X, Fan J, Ke AW (2020). Circular RNA circMET drives immunosuppression and anti-PD1 therapy resistance in hepatocellular carcinoma via the miR-30-5p/snail/DPP4 axis. Mol Cancer.

[55] Sun S, Wang W, Luo X, Li Y, Liu B, Li X, Zhang B, Han S, Li X (2019). Circular RNA circ-ADD3 inhibits hepatocellular carcinoma metastasis through facilitating EZH2 degradation via CDK1-mediated ubiquitination. Am J Cancer Res.

[56] Han D, Li J, Wang H, Su X, Hou J, Gu Y, Qian C, Lin Y, Liu X, Huang M (2017). Circular RNA circMTO1 acts as the sponge of microRNA-9 to suppress hepatocellular carcinoma progression. Hepatology.

[57] Xu L, Feng X, Hao X, Wang P, Zhang Y, Zheng X, Li L, Ren S, Zhang M, Xu M (2019). CircSETD3 (Hsa_circ_0000567) acts as a sponge for microRNA-421 inhibiting hepatocellular carcinoma growth. J Exp Clin Cancer Res.

[58] Zhu YJ, Zheng B, Luo GJ, Ma XK, Lu XY, Lin XM, Yang S, Zhao Q, Wu T, Li ZX (2019). Circular RNAs negatively regulate cancer stem cells by physically binding FMRP against CCAR1 complex in hepatocellular carcinoma. Theranostics.

[59] Liu B, Tian Y, Chen M, Shen H, Xia J, Nan J, Yan T, Wang Y, Shi L, Shen B (2021). CircUBAP2 promotes MMP9-mediated oncogenic effect via sponging miR-194-3p in hepatocellular carcinoma. Front Cell Dev Biol.

[60] Shi L, Liu B, Shen DD, Yan P, Zhang Y, Tian Y, Hou L, Jiang G, Zhu Y, Liang Y (2021). A tumor-suppressive circular RNA mediates uncanonical integrin degradation by the proteasome in liver cancer. Sci Adv.

[61] Memczak S, Jens M, Elefsinioti A, Torti F, Krueger J, Rybak A, Maier L, Mackowiak SD, Gregersen LH, Munschauer M (2013). Circular RNAs are a large class of animal RNAs with regulatory potency. Nature.

[62] Hansen TB, Jensen TI, Clausen BH, Bramsen JB, Finsen B, Damgaard CK, Kjems J (2013). Natural RNA circles function as efficient microRNA sponges. Nature.

[63] Salmena L, Poliseno L, Tay Y, Kats L, Pandolfi Pier P (2011). A ceRNA hypothesis: the Rosetta stone of a hidden RNA language?. Cell.

[64] Ding L, Zhao Y, Dang S, Wang Y, Li X, Yu X, Li Z, Wei J, Liu M, Li G (2019). Circular RNA circ-DONSON facilitates gastric cancer growth and invasion via NURF complex dependent activation of transcription factor SOX4. Mol Cancer.

[65] Du WW, Fang L, Yang W, Wu N, Awan FM, Yang Z, Yang BB (2017). Induction of tumor apoptosis through a circular RNA enhancing Foxo3 activity. Cell Death Differ.

[66] Hsu M-T, Coca-Prados M (1979). Electron microscopic evidence for the circular form of RNA in the cytoplasm of eukaryotic cells. Nature.

[67] Thomson DW, Dinger ME (2016). Endogenous microRNA sponges: evidence and controversy. Nat Rev Genet.

[68] Gebauer F, Schwarzl T, Valcarcel J, Hentze MW (2020). RNA-binding proteins in human genetic disease. Nat Rev Genet.

[69] Qin H, Ni H, Liu Y, Yuan Y, Xi T, Li X, Zheng L (2020). RNA-binding proteins in tumor progression. J Hematol Oncol.

[70] Abdelmohsen K, Panda AC, Munk R, Grammatikakis I, Dudekula DB, De S, Kim J, Noh JH, Kim KM, Martindale JL, Gorospe M (2017). Identification of HuR target circular RNAs uncovers suppression of PABPN1 translation by CircPABPN1. RNA Biol.

[71] Schneider T, Hung LH, Schreiner S, Starke S, Eckhof H, Rossbach O, Reich S, Medenbach J, Bindereif A (2016). CircRNA-protein complexes: IMP3 protein component defines subfamily of circRNPs. Sci Rep.

[72] Sun J, Gu X, Wu N, Zhang P, Liu Y, Jiang S (2018). Human antigen R enhances the epithelial-mesenchymal transition via regulation of ZEB-1 in the human airway epithelium. Respir Res.

[73] Kullmann M, Gopfert U, Siewe B, Hengst L (2002). ELAV/Hu proteins inhibit p27 translation via an IRES element in the p27 5'UTR. Genes Dev.

[74] Alpatov R, Lesch BJ, Nakamoto-Kinoshita M, Blanco A, Chen S, Stutzer A, Armache KJ, Simon MD, Xu C, Ali M (2014). A chromatin-dependent role of the fragile X mental retardation protein FMRP in the DNA damage response. Cell.

[75] Du WW, Zhang C, Yang W, Yong T, Awan FM, Yang BB (2017). Identifying and Characterizing circRNA-Protein Interaction. Theranostics.

[76] Li Q, Wang Y, Wu S, Zhou Z, Ding X, Shi R, Thorne RF, Zhang XD, Hu W, Wu M (2019). CircACC1 regulates assembly and activation of AMPK complex under metabolic stress. Cell Metab.

[77] Zeng Y, Du WW, Wu Y, Yang Z, Awan FM, Li X, Yang W, Zhang C, Yang Q, Yee A (2017). A circular RNA binds to and activates AKT phosphorylation and nuclear localization reducing apoptosis and enhancing cardiac repair. Theranostics.

[78] Yang Q, Du WW, Wu N, Yang W, Awan FM, Fang L, Ma J, Li X, Zeng Y, Yang Z (2017). A circular RNA promotes tumorigenesis by inducing c-myc nuclear translocation. Cell Death Differ.

[79] Yang ZG, Awan FM, Du WW, Zeng Y, Lyu J, Gupta S, Yang W, Yang BB (2017). The circular RNA interacts with STAT3, increasing its nuclear translocation and wound repair by modulating Dnmt3a and miR-17 function. Mol Ther.

[80] Pamudurti NR, Bartok O, Jens M, Ashwal-Fluss R, Stottmeister C, Ruhe L, Hanan M, Wyler E, Perez-Hernandez D, Ramberger E (2017). Translation of CircRNAs. Mol Cell.

[81] Meyer KD, Patil DP, Zhou J, Zinoviev A, Skabkin MA, Elemento O, Pestova TV, Qian SB, Jaffrey SR (2015). 5' UTR m(6)A promotes cap-independent translation. Cell.

[82] Chen C-Y, Sarnow P (1995). Initiation of protein synthesis by the eukaryotic translational apparatus on circular RNAs. Science.

[83] Legnini I, Di Timoteo G, Rossi F, Morlando M, Briganti F, Sthandier O, Fatica A, Santini T, Andronache A, Wade M (2017). Circ-ZNF609 Is a circular RNA that can be translated and functions in myogenesis. Mol Cell.

[84] Lien WH, Fuchs E (2014). Wnt some lose some: transcriptional governance of stem cells by Wnt/beta-catenin signaling. Genes Dev.

[85] Pang Y, Liu Z, Han H, Wang B, Li W, Mao C, Liu S (2020). Peptide SMIM30 promotes HCC development by inducing SRC/YES1 membrane anchoring and MAPK pathway activation. J Hepatol.

[86] Yao Z, Xu R, Yuan L, Xu M, Zhuang H, Li Y, Zhang Y, Lin N (2019). Circ_0001955 facilitates hepatocellular carcinoma (HCC) tumorigenesis by sponging miR-516a-5p to release TRAF6 and MAPK11. Cell Death Dis.

[87] He J, Huang Z, He M, Liao J, Zhang Q, Wang S, Xie L, Ouyang L, Koeffler HP, Yin D, Liu A (2020). Circular RNA MAPK4 (circ-MAPK4) inhibits cell apoptosis via MAPK signaling pathway by sponging miR-125a-3p in gliomas. Mol Cancer.

[88] Yang W, Du WW, Li X, Yee AJ, Yang BB (2016). Foxo3 activity promoted by non-coding effects of circular RNA and Foxo3 pseudogene in the inhibition of tumor growth and angiogenesis. Oncogene.

[89] Wang YG, Wang T, Ding M, Xiang SH, Shi M, Zhai B (2019). hsa_circ_0091570 acts as a ceRNA to suppress hepatocellular cancer progression by sponging hsa-miR-1307. Cancer Lett.

[90] Yang J, Antin P, Berx G, Blanpain C, Brabletz T, Bronner M, Campbell K, Cano A, Casanova J, Christofori G (2020). Guidelines and definitions for research on epithelial-mesenchymal transition. Nat Rev Mol Cell Biol.

[91] Giannelli G, Koudelkova P, Dituri F, Mikulits W (2016). Role of epithelial to mesenchymal transition in hepatocellular carcinoma. J Hepatol.

[92] Li X, Liu C-X, Xue W, Zhang Y, Jiang S, Yin Q-F, Wei J, Yao R-W, Yang L, Chen L-L (2017). Coordinated circRNA Biogenesis and Function with NF90/NF110 in Viral Infection. Mol Cell.

[93] Llovet JM, Montal R, Sia D, Finn RS (2018). Molecular therapies and precision medicine for hepatocellular Carcinoma. Nat Rev Clin Oncol.

[94] Kulik L, El-Serag HB (2019). Epidemiology and management of hepatocellular carcinoma. Gastroenterology.

[95] Chen J, Jin R, Zhao J, Liu J, Ying H, Yan H, Zhou S, Liang Y, Huang D, Liang X (2015). Potential molecular, cellular and microenvironmental mechanism of sorafenib resistance in hepatocellular carcinoma. Cancer Lett.

[96] Ruiz de Galarreta M, Bresnahan E, Molina-Sanchez P, Lindblad KE, Maier B, Sia D, Puigvehi M, Miguela V, Casanova-Acebes M, Dhainaut M (2019). Beta-catenin activation promotes immune escape and resistance to anti-PD-1 therapy in hepatocellular carcinoma. Cancer Discov.

[97] Tang W, Chen Z, Zhang W, Cheng Y, Zhang B, Wu F, Wang Q, Wang S, Rong D, Reiter FP (2020). The mechanisms of sorafenib resistance in hepatocellular carcinoma: theoretical basis and therapeutic aspects. Signal Transduct Target Ther.

[98] Wei L, Wang X, Lv L, Liu J, Xing H, Song Y, Xie M, Lei T, Zhang N, Yang M (2019). The emerging role of microRNAs and long noncoding RNAs in drug resistance of hepatocellular carcinoma. Mol Cancer.

[99] Lin Z, Xia S, Liang Y, Ji L, Pan Y, Jiang S, Wan Z, Tao L, Chen J, Lin C (2020). LXR activation potentiates sorafenib sensitivity in HCC by activating microRNA-378a transcription. Theranostics.

[100] Fan L, Huang X, Chen J, Zhang K, Gu Y-H, Sun J, Cui S-Y (2020). Long noncoding RNA MALAT1 contributes to sorafenib resistance by targeting miR-140-5p/Aurora—a signaling in hepatocellular carcinoma. Mol Cancer Ther.

[101] Yang C, Dong Z, Hong H, Dai B, Song F, Geng L, Lu J, Yang J, Sui C, Xu M (2020). circFN1 mediates sorafenib resistance of hepatocellular carcinoma cells by sponging miR-1205 and regulating E2F1 expression. Mol Ther Nucleic Acids.

[102] El-Serag HB, Rudolph KL (2007). Hepatocellular carcinoma: epidemiology and molecular carcinogenesis. Gastroenterology.

[103] Yamashita T, Koshikawa N, Shimakami T, Terashima T, Nakagawa M, Nio K, Horii R, Iida N, Kawaguchi K, Arai K, et al. Serum laminin gamma2 monomer as a novel diagnostic and predictive biomarker for hepatocellular carcinoma. *Hepatology* 2021. 10.1002/hep.31758 33609304

[104] Bahn JH, Zhang Q, Li F, Chan TM, Lin X, Kim Y, Wong DT, Xiao X (2015). The landscape of microRNA, Piwi-interacting RNA, and circular RNA in human saliva. Clin Chem.

[105] Kolling M, Haddad G, Wegmann U, Kistler A, Bosakova A, Seeger H, Hubel K, Haller H, Mueller T, Wuthrich RP, Lorenzen JM (2019). Circular RNAs in urine of kidney transplant patients with acute T cell-mediated allograft rejection. Clin Chem.

[106] Li Y, Zheng Q, Bao C, Li S, Guo W, Zhao J, Chen D, Gu J, He X, Huang S (2015). Circular RNA is enriched and stable in exosomes: a promising biomarker for cancer diagnosis. Cell Res.

[107] Wen G, Zhou T, Gu W. The potential of using blood circular RNA as liquid biopsy biomarker for human diseases. *Protein Cell* 2020. 10.1007/s13238-020-00799-3 PMC8674396 33131025

[108] Jia C, Yao Z, Lin Z, Zhao L, Cai X, Chen S, Deng M, Zhang Q (2021). circNFATC3 sponges miR-548I acts as a ceRNA to protect NFATC3 itself and suppressed hepatocellular carcinoma progression. J Cell Physiol.

[109] Herkt M, Thum T (2021). Pharmacokinetics and proceedings in clinical application of nucleic acid therapeutics. Mol Ther.

[110] Brown R, Curry E, Magnani L, Wilhelm-Benartzi CS, Borley J (2014). Poised epigenetic states and acquired drug resistance in cancer. Nat Rev Cancer.

[111] Choi YH, Yu AM (2014). ABC transporters in multidrug resistance and pharmacokinetics, and strategies for drug development. Curr Pharm Des.

[112] Zhang G, Huang X, Xiu H, Sun Y, Chen J, Cheng G, Song Z, Peng Y, Shen Y, Wang J, Cai Z (2020). Extracellular vesicles: natural liver-accumulating drug delivery vehicles for the treatment of liver diseases. J Extracell Vesicles.

[113] Bhaskaran V, Yao Y, Bei F, Peruzzi P (2019). Engineering, delivery, and biological validation of artificial microRNA clusters for gene therapy applications. Nat Protoc.

[114] Yang L, Han B, Zhang Z, Wang S, Bai Y, Zhang Y, Tang Y, Du L, Xu L, Wu F (2020). Extracellular vesicle-mediated delivery of circular RNA SCMH1 promotes functional recovery in rodent and nonhuman primate ischemic stroke models. Circulation.

[115] Burslem GM, Crews CM (2020). Proteolysis-targeting chimeras as therapeutics and tools for biological discovery. Cell.

[116] Huang C, Liang D, Tatomer DC, Wilusz JE (2018). A length-dependent evolutionarily conserved pathway controls nuclear export of circular RNAs. Genes Dev.

[117] Li S, Li X, Xue W, Zhang L, Yang LZ, Cao SM, Lei YN, Liu CX, Guo SK, Shan L (2021). Screening for functional circular RNAs using the CRISPR-Cas13 system. Nat Methods.

[118] Guo JU, Agarwal V, Guo H, Bartel DP (2014). Expanded identification and characterization of mammalian circular RNAs. Genome Biol.

[119] Hansen TB, Wiklund ED, Bramsen JB, Villadsen SB, Statham AL, Clark SJ, Kjems J (2011). miRNA-dependent gene silencing involving Ago2-mediated cleavage of a circular antisense RNA. EMBO J.

[120] Liu CX, Li X, Nan F, Jiang S, Gao X, Guo SK, Xue W, Cui Y, Dong K, Ding H (2019). Structure and degradation of circular RNAs regulate PKR activation in innate immunity. Cell.

[121] Li J, Qin X, Wu R, Wan L, Zhang L, Liu R (2020). Circular RNA circFBXO11 modulates hepatocellular carcinoma progress and oxaliplatin resistance through miR-605/FOXO3/ABCB1 axis. J Cell Mol Med.

